# High-Throughput Screening of Thiol–ene Click Chemistries for Bone Adhesive Polymers

**DOI:** 10.1021/acsami.3c12072

**Published:** 2023-10-31

**Authors:** Kavya Ganabady, Nicola Contessi Negrini, Jacob C. Scherba, Brandon M. Nitschke, Morgan R. Alexander, Kyle H. Vining, Melissa A. Grunlan, David J. Mooney, Adam D. Celiz

**Affiliations:** † Department of Bioengineering, Imperial College London, London W12 0BZ, U.K.; ‡ Wyss Institute for Biologically Inspired Engineering and Harvard John A. Paulson School of Engineering and Applied Sciences, Harvard University, Cambridge, Massachusetts 02138, United States; § Department of Biomedical Engineering, Texas A&M University, College Station, Texas 77843-3120, United States; ∥ School of Pharmacy, University of Nottingham, Nottingham NG7 2RD, U.K.; ⊥ School of Dental Medicine and Department of Materials Science, School of Engineering and Applied Science, University of Pennsylvania, Philadelphia, Pennsylvania 19104-6030, United States; # Francis Crick Institute, London NW1 1AT, U.K.

**Keywords:** high-throughput screening, click chemistry, cytocompatibility, bone adhesive, orthopedics

## Abstract

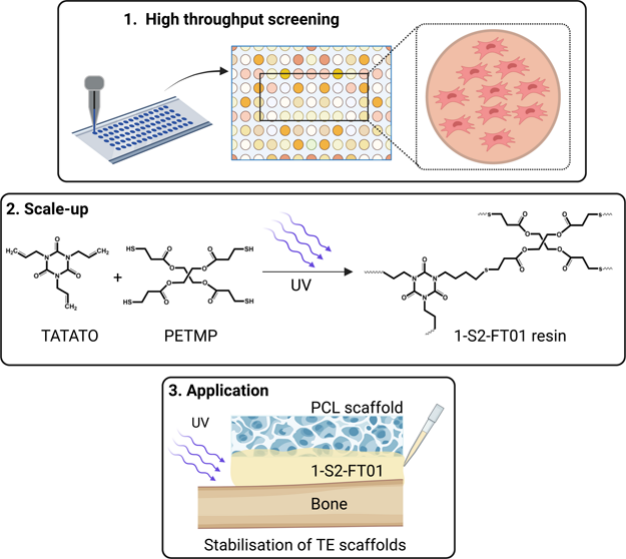

Metal surgical pins and screws are employed in millions of orthopedic surgical procedures every year worldwide, but their usability is limited in the case of complex, comminuted fractures or in surgeries on smaller bones. Therefore, replacing such implants with a bone adhesive material has long been considered an attractive option. However, synthesizing a biocompatible bone adhesive with a high bond strength that is simple to apply presents many challenges. To rapidly identify candidate polymers for a biocompatible bone adhesive, we employed a high-throughput screening strategy to assess human mesenchymal stromal cell (hMSC) adhesion toward a library of polymers synthesized via thiol–ene click chemistry. We chose thiol–ene click chemistry because multifunctional monomers can be rapidly cured via ultraviolet (UV) light while minimizing residual monomer, and it provides a scalable manufacturing process for candidate polymers identified from a high-throughput screen. This screening methodology identified a copolymer (1-S2-FT01) composed of the monomers 1,3,5-triallyl-1,3,5-triazine-2,4,6(1*H*,3*H*,5*H*)-trione (TATATO) and pentaerythritol tetrakis (3-mercaptopropionate) (PETMP), which supported highest hMSC adhesion across a library of 90 polymers. The identified copolymer (1-S2-FT01) exhibited favorable compressive and tensile properties compared to existing commercial bone adhesives and adhered to bone with adhesion strengths similar to commercially available bone glues such as Histoacryl. Furthermore, this cytocompatible polymer supported osteogenic differentiation of hMSCs and could adhere 3D porous polymer scaffolds to the bone tissue, making this polymer an ideal candidate as an alternative bone adhesive with broad utility in orthopedic surgery.

## Introduction

The number of new bone fractures in 2019 was estimated at 178 million cases globally.^1^ Surgical treatment of fractures is based on metal implants such as screws, plates, and Kirschner wires.^2^ These implants are generally made of stiff, bioinert metals, which present several limitations, such as stress shielding and lack of integration with the host tissue, leading to subsequent susceptibility to rejection and failure due to the body’s immune response, impacting patients’ healing and mobility, and potentially causing serious long-term damage.^3,4^ Moreover, as fracture complexity increases, the complexity of the surgery also increases. This is particularly relevant in fractured brittle bones of elderly patients and smaller bones with comminuted fractures where no fixation plates for fragments of this size are available.^5^ In such cases, bone adhesives represent an optimal alternative surgical approach, as they would decrease surgery time and improve surgical outcomes, particularly if the adhesive has mechanical properties similar to bones and is able to integrate with the host tissue.^6^ This has been widely researched, with commercially available biomaterial adhesives being developed for numerous other tissue types.^7,8^ However, due to the high mechanical strength requirements of the bone tissue, developing a polymeric bone adhesive that is biocompatible, adhesive to the bone, and simple to apply in a surgical setting presents several challenges.^9^

Currently, to our knowledge, there are no commercially available products that fulfill all of the above criteria. Cyanoacrylate-based glues, such as Super Bonder and Histoacryl, demonstrate up to 1.2 MPa bone adhesion and are already approved for use in skin adhesion applications, but they are not yet approved for use in bones due to cytotoxicity concerns.^10^ Other approaches, such as calcium–phosphate cements, offer strong adhesion to bone and simultaneously offer good biocompatibility.^6^ For example, Tetranite is a calcium–phosphate-based cement that has been validated in vivo to show a 1 MPa adhesive bond to bones and demonstrates total resorption after 4 months. This approach is currently under investigation in preclinical and clinical trials.^11^

To identify candidate polymers as biocompatible bone adhesives, we employed a high-throughput screening strategy to survey a library of polymers, focusing on the ability of the polymers to allow human mesenchymal stromal cell (hMSC) adhesion as the primary screening criterion. The choice of this criterion was primarily to ensure that the identified lead candidate polymers would demonstrate favorable biocompatibility in contrast with existing cyanoacrylate-based glues. Furthermore, cell adhesion assays have been proven to be a simple method to rapidly screen a high number of samples in parallel to identify promising materials for further development.^12^ hMSCs are adult mesenchymal progenitor cells that can differentiate into osteoblasts and adipocytes that reside in the bone marrow niche. A total of 90 different copolymers were synthesized by combining 15 acrylate, methacrylate, or allyl multifunctional monomers with 2 multithiol monomers in different stoichiometric ratios (Table S1), which we subsequently printed as a polymer microarray. Upon UV irradiation, the free-radical polymerization reaction occurred in a step-growth fashion, making use of thiol–ene click chemistry to rapidly form a polymer network.^13^ Click chemistries are a set of reactions that are rapidly processed in mild reaction conditions, have high yield, produce minimal byproducts and stable products, are simple to perform, and are insensitive to moisture and oxygen,^14^ making them useful for the design of biomaterials, which can be used in a clinical setting.

## Results and Discussion

**Figure 1:**
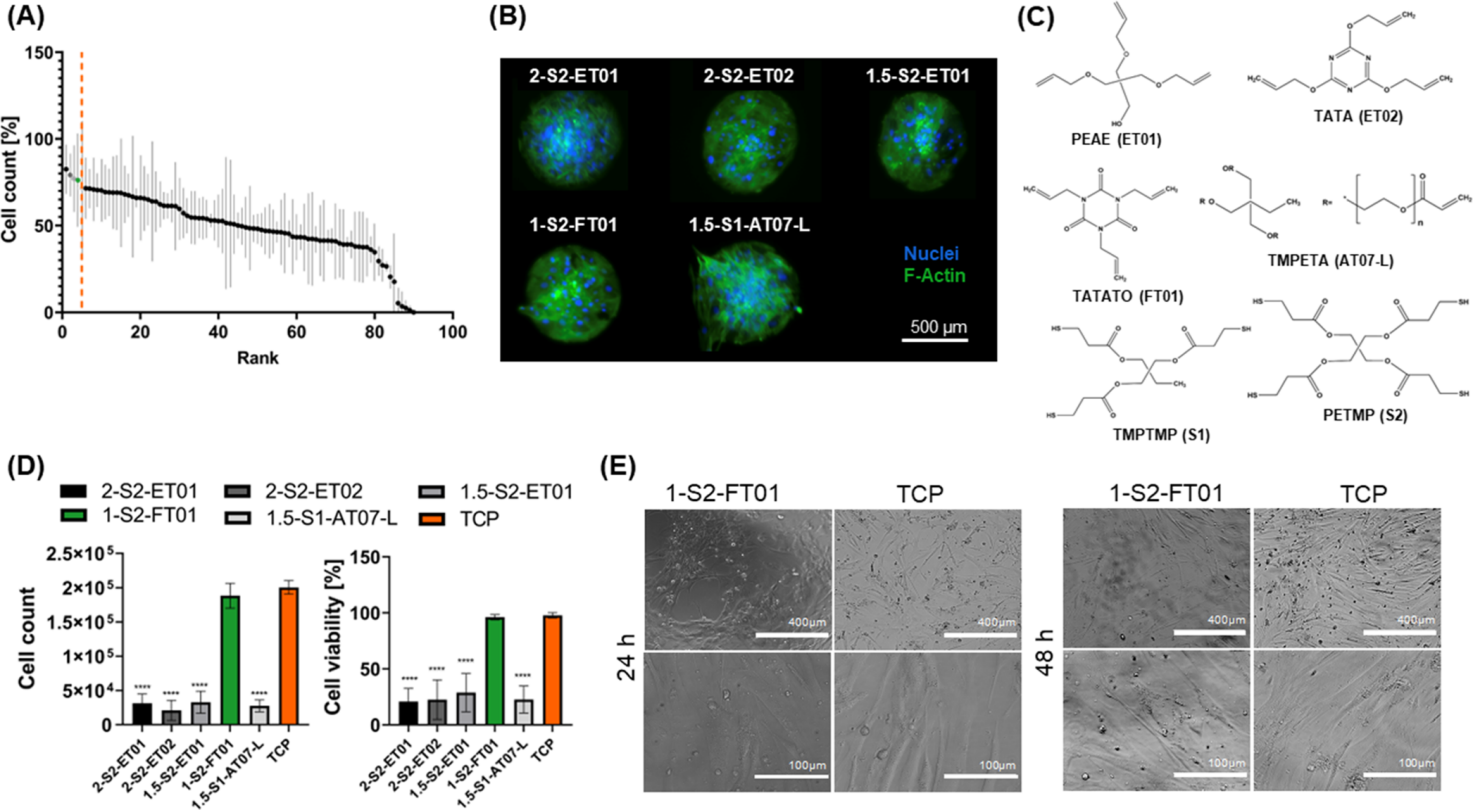
High-throughput screening of thiol–ene polymers via microarrays. (A) Quantification of the number of DAPI-stained hMSC nuclei on each copolymer. The grand mean was computed, and those copolymers whose counts were greater than 1.5 standard deviations above the grand mean are shown on the left of the orange line. The histogram shows the mean for each polymer chemistry ± standard deviation (*n* = 6 per chemistry). (B) Fluorescent imaging of lead copolymers on a microarray shows F-actin (green) and nuclei (blue). (C) Chemical structures of pentaerythritol allyl ether (PEAE (ET01)), 2,4,6-triallyloxy-1,3,5-triazine (TATA (ET02)), TATATO (FT01), trimethylolpropane ethoxylate triacrylate (TMPETA (AT07-L)), trimethylolpropane tris(3-mercaptopropionate) (TMPTMP (S1)), and PETMP (S2) monomers used for synthesis of the top 5-ranked hMSC adhesive copolymers. (D) hMSC count and viability on candidate copolymers vs. TCP controls (*n* = 8; ****p < 0.0001). (E) Phase microscopy images of hMSCs seeded on 1-S2-FT01 (TATATO/PETMP) and the TCP control for 24 and 48 h.

We used time-of-flight secondary-ion mass spectrometry (ToF-SIMS) to analyze the quality of the microarray printing and confirm that there was no leakage or leaching of monomers after printing and polymers within the microarray were discrete from one another (Figure S1).^15^ We then cultured hMSCs with the thiol–ene polymer microarray via an adhesion assay and ranked each polymer by the mean number of DAPI-stained hMSC nuclei (Figure 1A). The highest-ranked polymers supported the adhesion of viable cells, as shown by the presence of cells adhered on the polymer surface (Figure 1B). Lead candidate polymers were identified (*n* = 5) and scaled up into 24 well plates for further cytocompatibility testing (Figure 1C). Among these polymers, 1-S2-FT01, consisting of a 1:1 stoichiometric molar ratio of 1,3,5-triallyl-1,3,5-triazine-2,4,6(1*H*,3*H*,5*H*)-trione (TATATO) and pentaerythritol tetrakis (3-mercaptopropionate) (PETMP), showed higher cell adhesion and viability compared to the other polymers (*p* < 0.05, Figure 1D), and supported cell adhesion and viability comparable to the tissue culture plastic (TCP) control over 24 and 48 h (*p* > 0.05, Figures 1D,1E). Therefore, 1-S2-FT01 was chosen for further testing of its mechanical and biological properties, as well as suitability as a polymeric bone adhesive.

**Figure 2:**
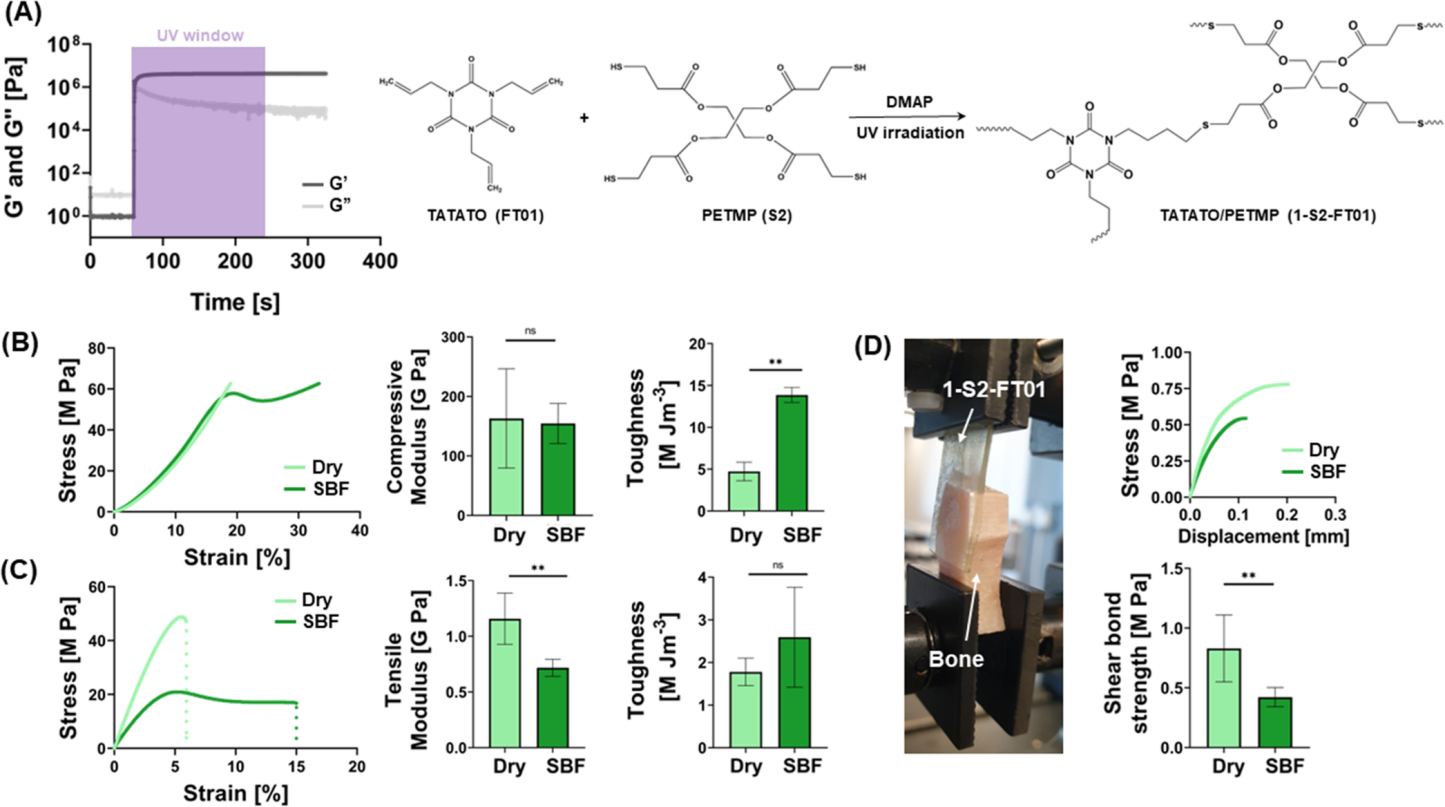
1-S2-FT01 resin mechanical characterization and bone adhesion tests. (A) Rheological characterization of 1-S2-FT01 curing when exposed to UV light (3 min UV light, 20 mW cm^–2^). (B) Compressive and (C) tensile properties of dry and SBF-soaked 1-S2-FT01 samples. (*n* = 5; **p < 0.01). (D) 1-S2-FT01 adhesion to bovine tibia segments. The representative image of the polymer adhered to the bone (left) and lap-shear tests of dry and SBF-soaked 1-S2-FT01 to the bone (right) (*n* = 6; **p < 0.01).

Rheological characterization was conducted to examine the cross-linking kinetics of the 1-S2-FT01 resin (Figure 2A). Before UV exposure, the monomer mixture was a viscous solution characterized by a prevalent loss modulus (*G*″) and lower storage modulus (*G*′). Polymerization occurred immediately after UV exposure, with a near-instantaneous crossover of *G*′ and *G*″ and a plateau in *G*′ in 5 s, indicating the rapid nature of this curing method. The reaction begins with the formation of a thiyl radical through hydrogen exchange between the thiol group of PETMP and the radical source (2,2-dimethoxy-2-phenylacetophenone). The thiyl radical reacts rapidly with the vinyl group of TATATO, forming a thio–ether covalent bond and transferring the radical to the next thiol group.^16^ The rapid curing is due to the highly efficient free-radical chain-transfer reaction that occurs during the photopolymerization of thiol–ene polymers.^17^ This fast reaction provides not only a convenient curing time for surgery but also negates excessive UV exposure, which can potentially minimize cellular damage during surgery.^9^ Using Fourier transform infrared spectroscopy (FTIR), Lu et al. calculated the ultimate functional group conversion for the allyl and thiol as 91 and 85%, respectively; this difference in the final conversion is likely caused by a small amount of homopolymerization that occurs with the allyl functional group, as the allyl carbon radical propagates slowly through the allyl double bond.^13^ The minimal homopolymerization ensures an ordered structure of repeating TATATO/PETMP units, allowing for a more homogeneous network formation.

Compressive and tensile mechanical tests were carried out on the 1-S2-FT01 resin in both dry and SBF-soaked conditions (21 days immersion) to mimic conditions following initial adhesion to bone and after adhesion in prolonged physiological conditions (Figure 2B,2C). 1-S2-FT01 was characterized by a compressive modulus of approximately 162.9 ± 83.3 MPa when dry and 154.6 ± 33.5 in SBF-soaked conditions (Figure 2B), both of which are above the threshold stated for bone adhesives.^9^ The toughness of the resin increased after immersion in SBF, which is likely due to water absorption and plasticity increasing over time.^18^ The tensile modulus of the 1-S2-FT01 resin was 1.2 ± 0.2 GPa when dry and 0.7 ± 0.1 GPa after SBF soaking, compared to 114 GPa for the Ti6Al4 V titanium alloy commonly used in metal implants.^19^ This is significantly closer to Young’s modulus of the native bone (from 0.4 GPa for the trabecular bone to 17.9 GPa for the cortical bone), indicating that the 1-S2-FT01 resin may induce less stress shielding, thereby reducing postoperative bone resorption and potential refracture.^20,21^ However, the achieved Young’s modulus is significantly lower than that of the cortical bone, potentially limiting the application of 1-S2-FT01 to comminuted fractures and non-load-bearing sites.^20^ The tensile modulus significantly decreased after soaking in SBF, and despite no differences in toughness, the stress–strain curve clearly indicated a greater plasticity after prolonged immersion in SBF. In fact, dry samples showed a brittle tensile response, breaking almost immediately after reaching maximum stress, whereas there was a pronounced necking region in the SBF-soaked sample. This increase in plasticity was evidenced by a decrease in the maximum tensile stress and an increase in the maximum tensile strain after SBF immersion (Figure S2).

The 1-S2-FT01 resin adhered well to the bone (Figure 2D). The force required to detach the resin from bone was 0.8 ± 0.3 MPa, which is only slightly lower than commercially available bone adhesives, such as Super Bonder or Histoacryl (1.16 and 1.22 MPa respectively), and it surpassed the generally agreed threshold of 0.2 MPa minimum shear adhesive strength.^10,22^ Even after immersion in SBF for 3 weeks, 1-S2-FT01 remained adhered to the bone. Despite the reduction in adhesive strength from 0.8 ± 0.3 to 0.4 ± 0.1 MPa after 3 weeks of immersion in SBF, it remained above the minimum threshold.

A similar polymer chemistry, TATATO/TEMPIC (tris[2-(3-mercapto propionyloxy)ethyl] isocyanurate), has been previously studied as part of a fiber-reinforced composite combined with self-etching dental primers for bone adhesion, exhibiting a high bond strength of 9.0 MPa.^23^ However, with no primer, fillers, or fiber reinforcement, the shear bond strength was reduced to just 0.12 MPa, representing a 6-fold lower adhesion strength than the 1-S2-FT01 resin. Additionally, the preparation of a fiber-reinforced patch is complex and results in a device that cannot be applied easily in a surgical setting. In contrast, the 1-S2-FT01 resin is an in situ curable bone adhesive that achieves bond strengths in the MPa range, comparable to commercially available bone glues, without the need for primers, composites, or fiber reinforcement.

**Figure 3:**
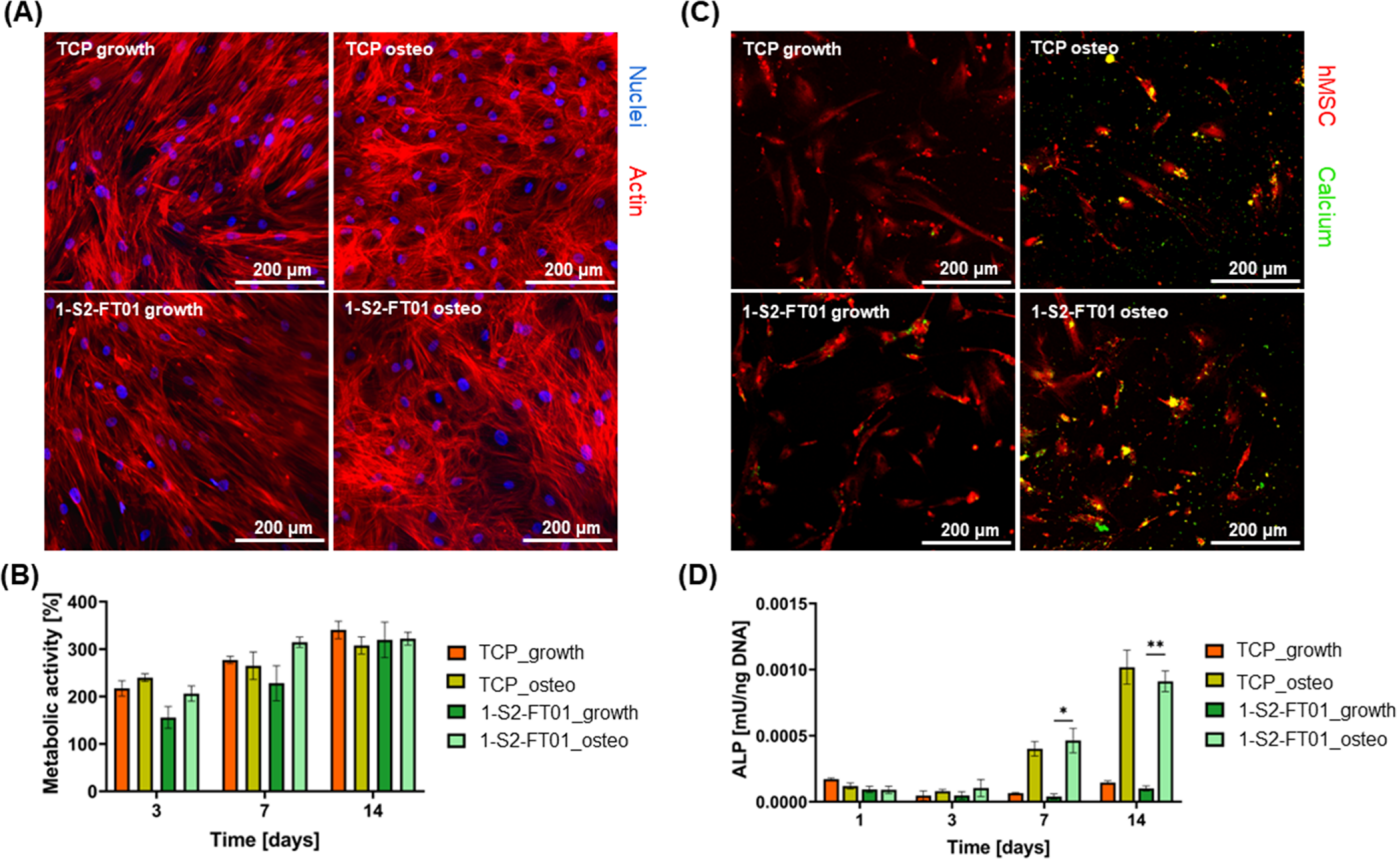
In vitro cytocompatibility and osteogenic differentiation analyses. (A) Representative confocal microscopy images of hMSCs cultured on 1-S2-FT01 and TCP in growth (left) and differentiation (right) media (Hoechst and ActinRed staining). (B) Relative metabolic activity of hMSCs cultured in proliferation (growth) and osteogenic (osteo) media, seeded directly on 1-S2-FT01 and TCP, as the control (*n* = 6). (C) Representative confocal microscopy images of hMSCs cultured on 1-S2-FT01 and TCP in growth (left) and differentiation (right) media (calcein and CellTracker deep red staining). (D) ALP expression normalized on DNA content (*n* = 6; **p < 0.01).

Finally, we conducted more in-depth in vitro studies to verify the cytocompatibility of the 1-S2-FT01 resin for its potential application as a bone adhesive (Figure 3).

Indirect cytotoxicity tests (Figure S3) showed the absence of indirect cytotoxicity of 1-S2-FT01 before and after curing since the viability of cells cultured in the presence of 1-S2-FT01 eluates was comparable to that of TCP controls over the standard 24 and 72 h incubation periods. Hoechst and ActinRed 555 staining of hMSC cultures seeded directly on the 1-S2-FT01 surface showed healthy cell morphology and confluency in both osteogenic and nonosteogenic media comparable to analogous conditions on TCP (Figure 3A). 2D culture of hMSCs on 1-S2-FT01 showed increased metabolic activity over a 14-day culture period (Figure 3B). The percentage increase in cell viability normalized on day 1 was comparable for 1-S2-FT01 and the TCP control. The ability to promote cell adhesion is a valuable biomaterial property, as cell attachment and proliferation on the surface of the biomaterial have been shown to decrease the likelihood of fibrous encapsulation.^24^ Direct contact between the bone and an implant is crucial in preventing fibrous encapsulation, so these substrates were also tested for their ability to support hMSC differentiation. Calcein and CellTracker deep red staining of hMSC cultures showed calcium deposition in 1-S2-FT01 and TCP in osteogenic media, qualitatively confirming cell osteogenic differentiation (Figure 3C).^25,26^ The expression of alkaline phosphatase, an early osteogenic differentiation marker, was found to be comparable for the duration of the in vitro culture for cells cultured on 1-S2-FT01 and TCP (Figure 3D). Taken together, these data suggest that osteogenic differentiation of cells adhered to the 1-S2-FT01 surface and that the 1-S2-FT01 resin has the potential to form a structural, functional bond between the implant and bone.^21,27^

**Figure 4:**
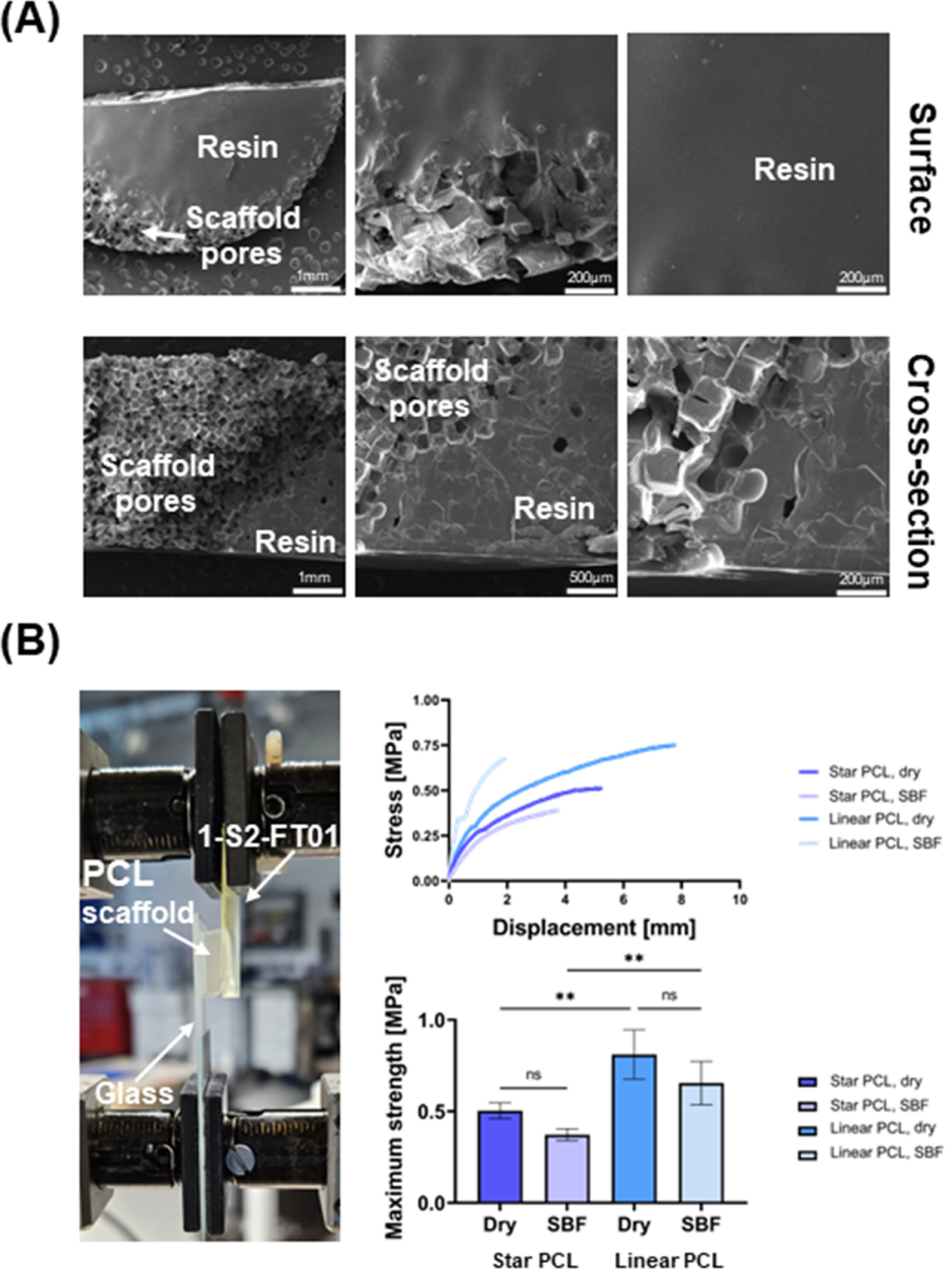
1-S2-FT01 adhesion to *star*- and *linear*-PCL scaffolds. (A) Representative image of lap-shear adhesion testing of 1-S2-FT01 to a *linear*-PCL scaffold (dry) (left). For dry and SBF-soaked 1-S2-FT01 to PCL scaffold constructs, stress versus displacement curves of lap-shear adhesion tests (top right) and maximum strength achieved in lap-shear tests (bottom right, *n* = 6; **p < 0.01). (B) Representative scanning electron microscopy (SEM) images of 1-S2-FT01/*linear*-PCL scaffold adhesion showing the construct’s surface (top row) and the cross-section (bottom row).

To assess the 1-S2-FT01 resin’s ability to adhere tissue engineering scaffolds to the bone, we used shape memory polymer (SMP) ε-polycaprolactone (PCL) scaffolds as an example. Both *star*-PCL (*T*_m_ = ∼45 °C, compressive modulus = ∼3.57 MPa) and *linear*-PCL (*T*_m_ = ∼55 °C; compressive modulus = ∼9.65 MPa) scaffolds have been produced from the corresponding acrylated macromers, yielding scaffolds with the ability to undergo press-fitting and shape recovery within a defect after exposure to temperatures above their melt transition (*T*_m_).^28^ These bone-regenerative PCL scaffolds also possess biodegradability and pore interconnectivity (pore diameter ∼220 μm) to facilitate osteoinduction but do not possess any adhesive properties, which limits their usability for the treatment of confined bone defects.^29,30^ Scanning electron microscopy (SEM) images show that the 1-S2-FT01 resin penetrates 1 mm into the PCL scaffolds, causing physical interlocking of the PCL and resin and contributing to a strong adhesive bond (Figure 4A). PCL scaffolds adhered to the 1-S2-FT01 resin with maximum strengths in the MPa range (Figure 4B). The lap-shear tests resulted in cohesive failure of *star-* and *linear*-PCL scaffolds, both dry and after SBF soaking; therefore, the adhesion strength could not be quantified. However, these results showed that the adhesion strength was at least 0.49 ± 0.05 MPa for *star*-PCL scaffolds and 0.74 ± 0.10 MPa for *linear*-PCL scaffolds, which is comparable to the adhesion strength of the 1-S2-FT01 resin to the bone, confirming the utility of the resin to bond such scaffolds to the bone (Supporting Video 1). These results present several promising avenues for further research using the 1-S2-FT01 resin as an adhesive material to support bone tissue engineering scaffolds.

## Conclusions

We have identified the 1-S2-FT01 resin through high-throughput screening of a library of thiol–ene polymers as a promising biocompatible bone adhesive. This resin was identified via favorable hMSC adhesion properties and displayed compressive and tensile moduli similar to the bone, even after 3 weeks of immersion in SBF. Its simple formulation and short curing time make it optimal for application in a surgical setting. The adhesive strength of the 1-S2-FT01 resin is well-suited for adhesion of comminuted or non-load-bearing fractures, and the adhesion strength is in the same order of magnitude as commercially available bone glues. This polymer adhesive may lead to shorter operation times, better integration, and reduced likelihood of stress shielding compared to metal implants, making this approach very appealing for complex fractures in small or brittle bones. As with other existing polymeric bone adhesives, we do not expect 1-S2-FT01 to degrade in vivo. However, its ability to bond to polymeric tissue engineering scaffolds widens its scope as a fixative material, allowing it to be combined with bone-regenerative approaches for enhanced applicability.

## Methods

All materials were purchased from Sigma-Aldrich unless otherwise specified.

### High-Throughput Screening

Polymeric microarrays were prepared as described by Vining et al.^31^ Briefly, 90 copolymers made from commercially available monomers were printed (250–400 μm) and photopolymerized on polyHEMA coated slides in microarrays, with six replicates per array. hMSCs were seeded (75 000 cells per array) in serum-free media for 24 and 48 h. For scale-up, candidate monomers from the screen were mixed with a thiol–ene cross-linking reagent (PETMP or TMPTMP) and 1% w/v of the photoinitiator 2,2-dimethoxy-2-phenylacetophenone. In a typical 12-well dish, a mixed monomer solution (400 μL) was added to each well and irradiated with 365 nm UV irradiation for 3 min at an intensity of 20 mW cm^–2^. Polymer coatings were UV sterilized for 30 min and then washed three times with Hanks’ balanced salt solution (HBSS).

### 1-S2-FT01 Resin

The 1-S2-FT01 resin was synthesized by mixing the two monomers in a 1:1 stoichiometric molar ratio with 1w/v of the photoinitiator 2,2-dimethoxy-2-phenylacetophenone. The monomer mixture was placed and cured with 20 mW cm^–2^ UV light (Omnicure S1500) for 3 min.

### SBF

SBF was prepared as described by Meskinfam et al.^32^

### Bovine Bone Tissue

Commercially available bovine shin bone tissue was used for the bone adhesion experiments. Specimens were cut to a size (50 mm × 15 mm) just prior to use.

### Adhesion of 1-S2-FT01 to the Bone Tissue

Lap-shear test samples were prepared by filling molds with dimensions of 35 mm (length) × 15 mm (width) × 5 mm (thickness) with 1-S2-FT01. Precut bovine bone samples were placed on top of the molds, resulting in a contact area of 15 mm × 15 mm. The samples were then irradiated with 365 nm UV irradiation for 3 min at an intensity of 20 mW cm^–2^.

### Adhesion of PCL Scaffolds to the Bone Tissue with 1-S2-FT01

*Star-* and *linear*-PCL scaffolds were prepared per a prior report.^28^ Specimens were prepared with dimensions of 15 mm (length) × 5 mm (width) × 5 mm (thickness) using a vibratome and a single-edge razor blade. To adhere a scaffold to the bone tissue, 100 μL of resin was placed onto the bone, followed by a PCL scaffold. Samples were then irradiated with 365 nm UV irradiation for 3 min at an intensity of 20 mW cm^–2^.

### Mechanical Testing

Rheological time sweeps were conducted by using a Kinexus Netzsch Ultra+ rheometer to evaluate the polymerization kinetics of 1-S2-FT01. Compressive, tensile, and lap-shear tests were conducted on dry and SBF-soaked (21 days, 37 °C) samples using an Instron 5866 Universal testing machine (10 kN load cell). Compression samples were made according to ISO 604:2002 and tested at 1 mm min^–1^ with a 0.05 kN preload (*n* = 5). Tensile test dog-bone samples were made to ISO 527-1:2019 and tested at 1 mm min^–1^ with a 0.05 kN preload (*n* = 5). The elastic modulus was calculated as the slope of the stress–strain curve in the 0–2% strain region. Lap-shear tests of 1-S2-FT01 adhered to the bovine bone and PCL scaffolds adhered to the bovine bone with 1-S2-FT01 were carried out at 1 mm min^–1^.

### In Vitro hMSC Studies

Indirect cytotoxicity tests using conditioned media were conducted using 1-S2-FT01, TCP (negative control), and rubber (positive control), according to ISO 10993-5. Media was incubated for 24 and 72 h and then used to culture semiconfluent hMSCs (passage 4, obtained from an 18–25-year-old female donor with consent via Thermo Fisher Scientific). Cell viability was measured via an AlamarBlue assay (Thermo Fisher Scientific).

Direct cytocompatibility tests were performed by seeding hMSCs (75 000 cells cm^–2^). hMSCs were cultured in osteogenic (“osteo”) and nonosteogenic (“growth”) media on 1-S2-FT01 and TCP (control). Growth media were formulated using high glucose Dulbecco’s modified Eagle medium (DMEM) with 10% v/v fetal bovine serum and 1% v/v penicillin/streptomycin. The osteogenic medium was formulated using low glucose Dulbecco’s modified Eagle medium (DMEM) with 10% (v/v) fetal bovine serum, 1% (v/v) penicillin/streptomycin, 100 nM dexamethasone, 50 μM ascorbic acid, and 10 mM B-glycerophosphate. AlamarBlue was used to measure hMSC metabolic activity after 1, 3, 7, and 14 days of cell culture. The fluorescence (excitation: 530–560 nm; emission: 590 nm) measured at each time point (Clariostar Plus Microplate Reader) was normalized on the fluorescence at day 1 to calculate the percentage cell viability. Alkaline phosphatase (ALP) activity was measured using a commercially available kit (BioTechne Ltd.) and normalized on the intracellular DNA content quantified via a commercially available kit (Abcam) after 1, 3, 7, and 14 days. After 14 days of culture, hMSCs were fixed in paraformaldehyde prior to fluorescent staining using Hoechst and ActinRed 555. Calcein staining was conducted by adding calcein into the media (1 μM solution) and incubating cells for 2 days prior to confocal imaging. CellTracker deep red was added for 30 min prior to imaging to visualize cells.

### Statistical Analysis

Data are shown as the mean ± standard deviation. Statistical analysis was performed by Prism-GraphPad software. The normal distribution of data was checked by the Shapiro–Wilk test. Differences between data groups were investigated by either two-way ANOVA with Tukey’s multiple comparison or multiple unpaired *t*-tests. Statistical significance between data groups was set for *p* < 0.05.

## Supporting Information Available

Additional experimental details and methods; including graphs of further experiments (PDF)
Resin pcl adhesion video (MP4)
