# Supplementary material for: High-Throughput Screening of Thiol–ene Click Chemistries for Bone Adhesive Polymers
Source: ACS Appl Mater Interfaces. 2023 Oct 31;15(44):50908–15. doi: 10.1021/acsami.3c12072 (PMC10636719; doi:10.1021/acsami.3c12072)
Supplement: Supplementary file 1 — am3c12072_si_001.pdf [file am3c12072_si_001.pdf]

Supporting Information

High throughput screening of thiol-ene click chemistries for bone adhesive polymers

Authors: Kavya Ganabady[1], Nicola Contessi Negrini[1], Jacob C. Scherba[2], Brandon M. Nitschke[3] Morgan R. Alexander[4], Kyle H. Vining[5], Melissa A. Grunlan[3], David J. Mooney[2], Adam D. Celiz[1,6]*

** Corresponding author: a.celiz@imperial.ac.uk.*

Affiliations: [1]Department of Bioengineering, Imperial College London, [2]Wyss Institute, Harvard University, [3] Department of Biomedical Engineering, Texas A&M University, [4]School of Pharmacy, University of Nottingham, [5]School of Dental Medicine and Department of Materials Science, School of Engineering and Applied Science, University of Pennsylvania, USA, [6] Francis Crick Institute, London, UK.

Table of Contents

Table S1. Constituent monomers to synthesize polymers in high throughput screen.

Figure S1. TOF-SIMS analysis of polymer microarray.

Figure S2. Maximum tensile stress and maximum tensile strain for dry and SBF-soaked TATATO/PETMP samples.

Figure S3. Indirect cytotoxicity studies.

Table S1. Constituent monomers used to synthesize polymers in high throughput screen.

Code	Chemical name	Diagram
AT02	Trimethylolpropane propoxylate triacrylate, Mn: 644	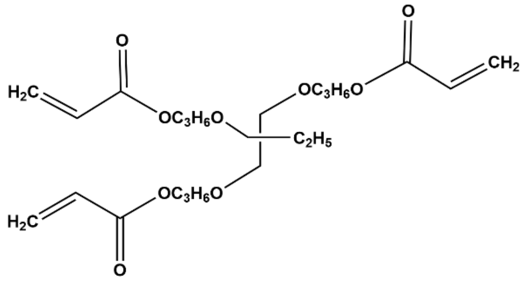
AT03	Trimethylolpropane triacrylate	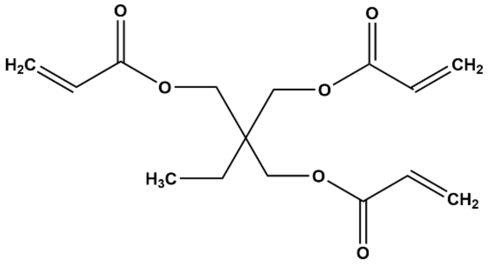
AT06	Pentaerythritol triacrylate	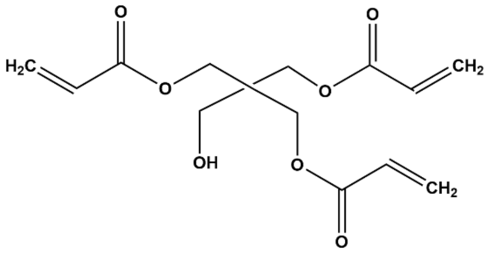
AT07-S	Trimethylolpropane ethoxylate triacrylate, Mn: 428	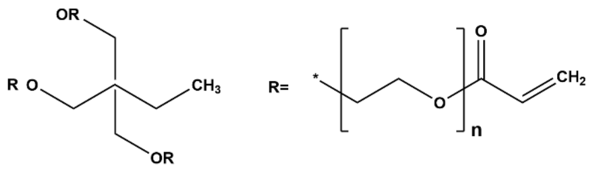
AT07-M	Trimethylolpropane ethoxylate triacrylate, Mn: 692	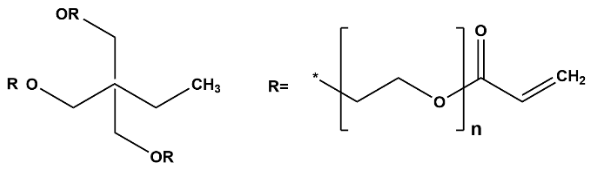

AT07-L	Trimethylolpropane ethoxylate triacrylate, Mn: 912	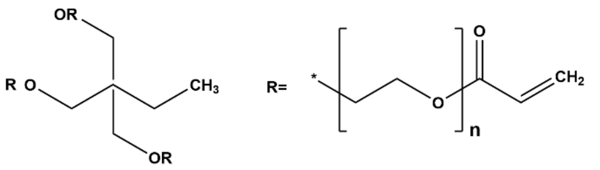
AT08	Glycerol propoxylate (1PO/OH) triacrylate	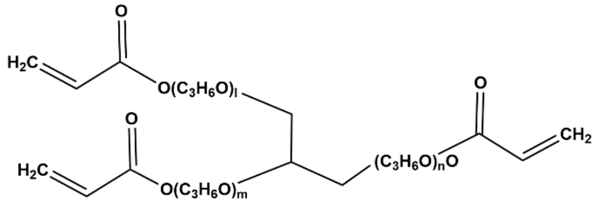
AT09	Tris[2-(acryloyloxy)ethyl] isocyanurate	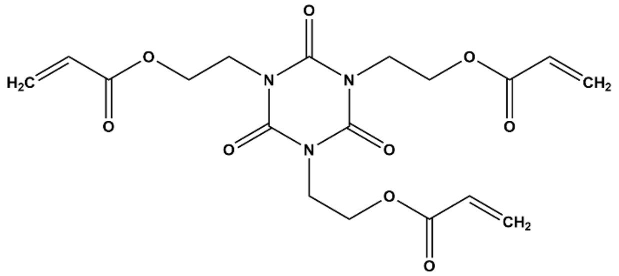
AT10	Zirconium bromonorbornanelactone carboxylate triacrylate	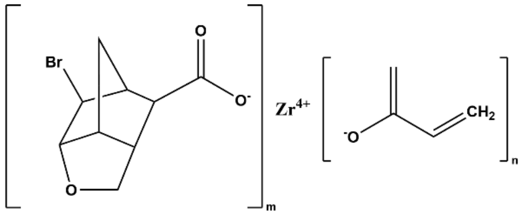
AT11	Di(trimethylolpropane) tetraacrylate	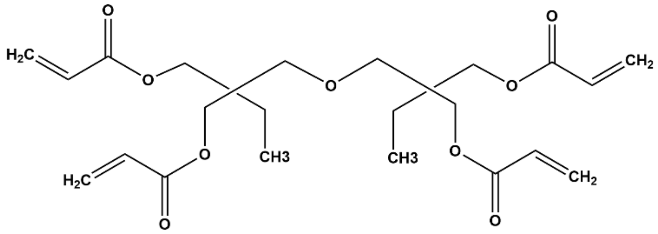

AT12	Pentaerythritol tetraacrylate	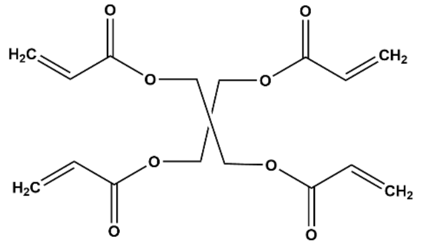
BT01	Trimethylolpropane trimethacrylate	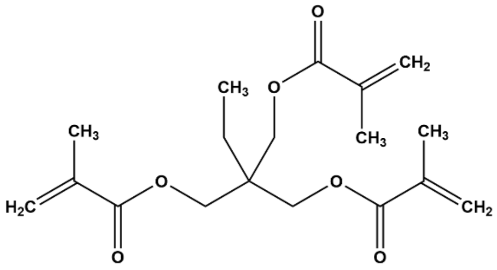
ET01	Pentaerythritol allyl ether	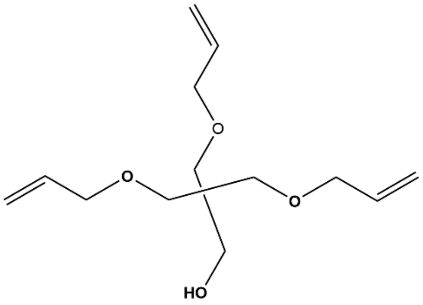
ET02	2,4,6-Triallyloxy-1,3,5-triazine	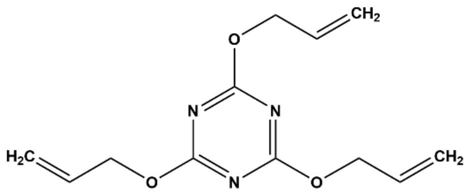
FT01	1,3,5-Triallyl-1,3,5-triazine-2,4,6(1H,3H,5H)-trione	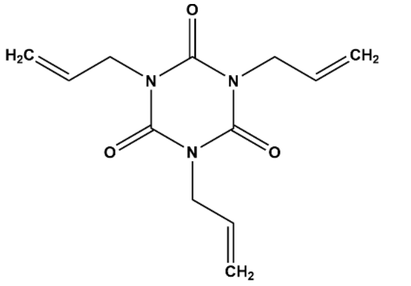

S1	Trimethylolpropane tris(3-mercaptopropionate)	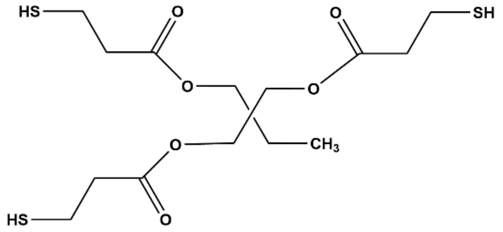
S2	Pentaerythritol tetrakis (3-mercaptopropionate)	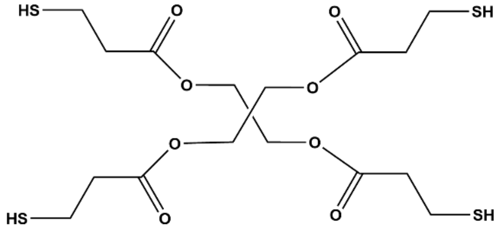

(A) Positive ion mode

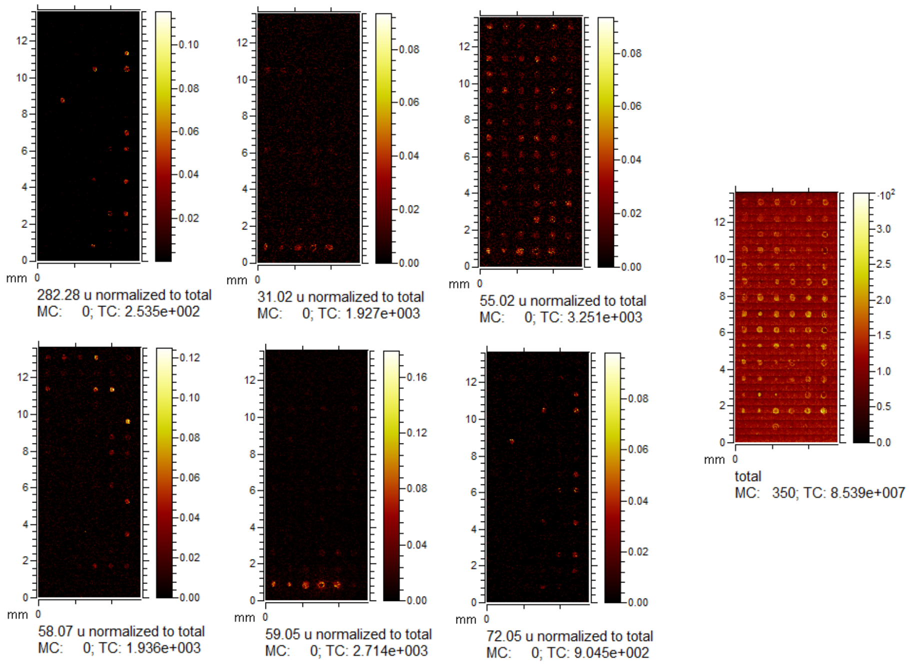

(B) Negative ion mode

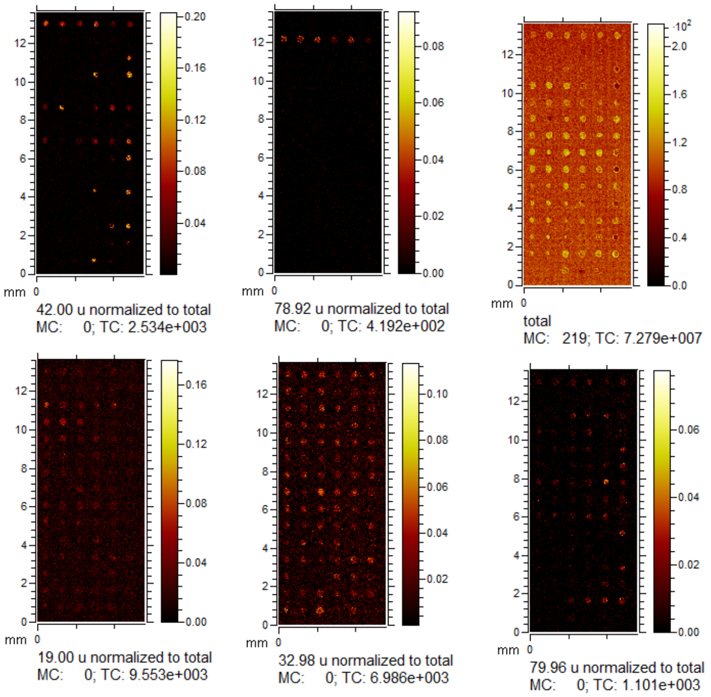

Figure S1. TOF-SIMS analysis of polymer microarray. ToF-SIMS measurements were conducted using a ToF-SIMS 4 (IONTOF GmbH) instrument operated using a 25 kV Bi_3^+ primary ion source exhibiting a pulsed target current of ~1 pA. Samples were scanned at a pixel density of 100 pixels per mm, with 8 shots per pixel over a given area. An ion dose of 2.45×10^{11} ions per cm^2 was applied to each sample area ensuring static conditions were maintained throughout. Both positive and negative secondary ion spectra were collected (mass resolution of >7000), over an acquisition period of 15 scans (the data from which were added together). Owing to the non-conductive nature of the samples, charge compensation was applied in the form of a low energy (20 eV) electron floodgun. Large area scans were taken from the entire polymer microarray and regions of interest were selected from the image to extract spectra from individual polymers. **(A)** Positive ion mode. (Left to right: 282.28 = $C_{18}H_{36}NO^+$; 31.02 = CH_3O^+; 55.02 = $C_3H_3O^+$; 58.07 = $C_3H_8N^+$; 59.05 = $C_3H_7O^+$; 72.05 = $C_3H_6NO^+$.)

(B) Negative ion mode. (Left to right: 42 = CNO^-; 78.92 = C_4HNO^-; 19 = F^-; 32.98 = HO_2^-; 79.96 = $C_4O_2^-$.)

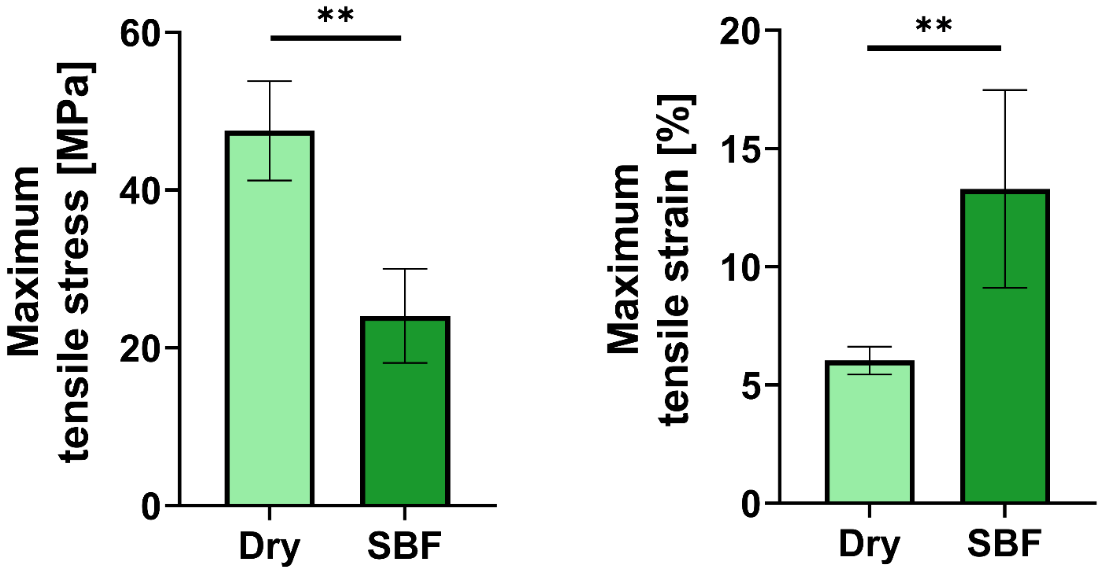

Figure S2. Maximum tensile stress and maximum tensile strain for dry and SBF-soaked 1-S2-FT01 resin. (n = 5; ** $p < 0.01$).

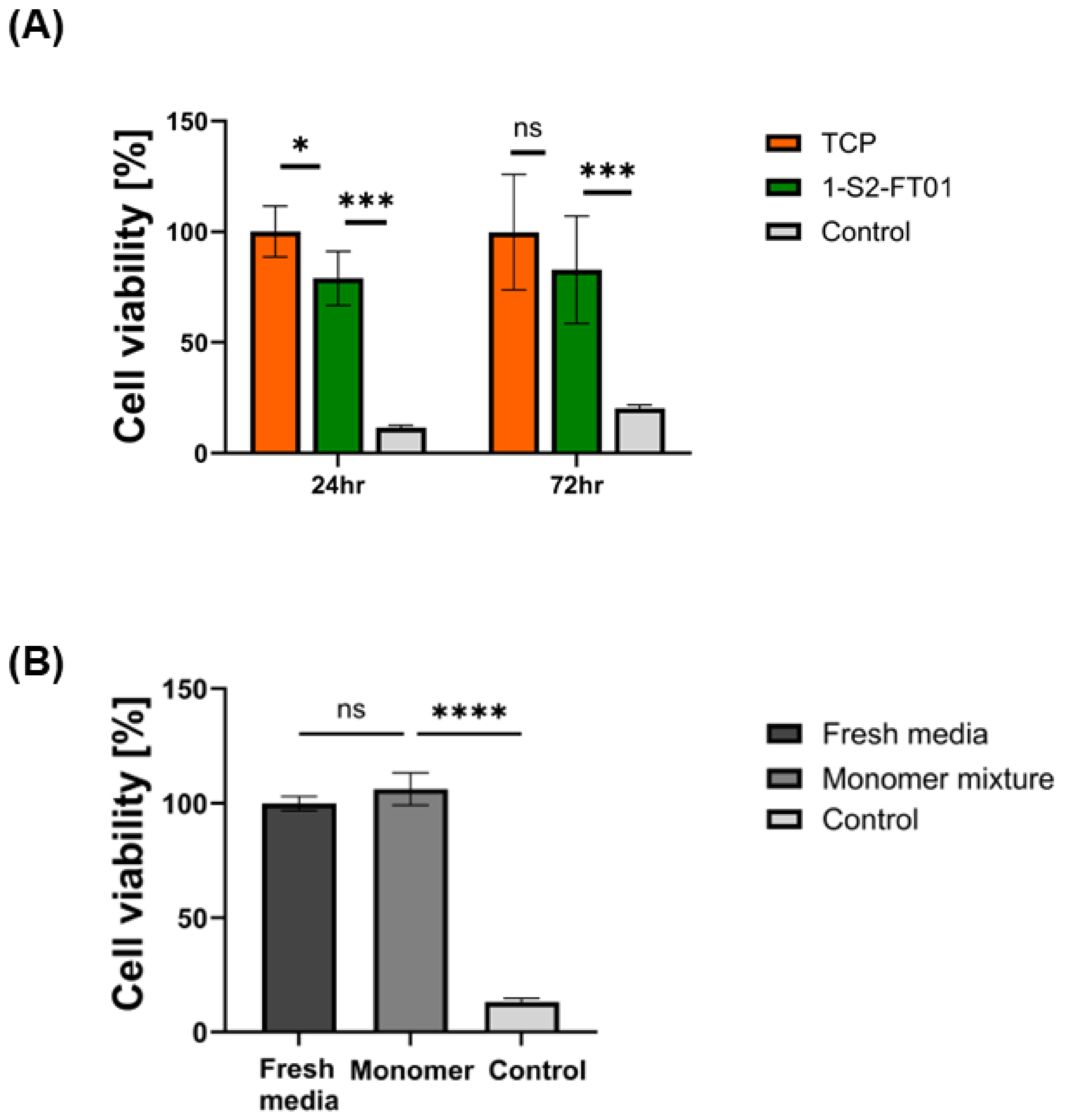

Figure S3. (A) Indirect cytotoxicity studies conducted using conditioned media (ISO 10993-5) in contact with 1-S2-FT01 resin for 24 and 72 h. Positive control: rubber, negative control: TCP. (n = 6; **** $p < 0.0001$). **(B)** Indirect cytotoxicity studies conducted using conditioned media (ISO 10993-5) in contact with uncured monomer mixture (TATATO, PETMP, 2,2-dimethoxy-2-phenylacetophenone). Media was conditioned for 5 minutes with the monomer mixture to mimic the surgical application time before the polymer is fully crosslinked. hMSCs were in contact with

conditioned media for 24 h. Positive control: rubber, negative control: fresh media. (n = 6; **** $p < 0.0001$).
